# Supplementary material for: K103N, V106M and Y188L Significantly Reduce HIV-1 Subtype C Phenotypic Susceptibility to Doravirine
Source: Viruses. 2024 Sep 20;16(9):1493. doi: 10.3390/v16091493 (PMC11437401; doi:10.3390/v16091493)
Supplement: Supplementary file 1 [file viruses-16-01493-s001.zip › viruses-3204800-supplementary.pdf]

Table S1: HIV-1 subtype B and C laboratory-adapted strains.

HIV-1 Subtype	Strain	GeneBank accession number	Publication
B	DS9	MH234640	Obasa *et al*., 2019 [65]
	LTNP5	AY835779	Mikhail *et al*., 2005 [66]
	SM1	KY060873	Ledwaba *et al*., 2019 [67]
	SM2	KF469956	Maldarelli *et al*., 2013 [68]
C	CM9	AF411967	Papathanasopoulos et al., 2002 [69]
	DU151	AF544009	Williamson *et al*., 2003 [70]
	DU179	AY043174	van Harmelen *et al*., 2001 [71]
	DU422	AF544005	Williamson *et al*., 2003 [70]

Table S2: SDM primers used to generate the single and combination NNRTI profiles.

DRM Profile	Primer Name*	Mutation	Sequence (5' → 3')**	optimised T_m (°C)
A98G, K103N, P225H	A98G, K103N forward	A98G	AGGGTTAAAAAAGAATAAATCAGTAACAGTATTAG	63
A98G, K103N	A98G, K103N reverse		CCTGGGTGCGGTATTCCTAATTGAAC	63
L100I, K103N	L100I, K103N forward	L100I	AAAAAAGAATAAATCAGTAACAGTATTAGATGTG	56.9
L100I,K103N,P225H	L100I, K103N reverse		ATCCCTGCTGGGTGCGGTATTCCTAA	55.1
K101P,K103N	K101P, K103N forward	K101P	AAAGAATAAATCAGTAACAGTATTAGATGTGGGG	61.3
	K101P, K103N reverse		GGTAACCCTGCTGGGTGCGGTATTCC	
K103N,V106M,F227L	K103N, V106M forward	K103N	TAAATCAATGACAGTATTAGATGTGGGGGATG	63
K103N, V106M	K103N, V106M reverse	V106M	TTCTTTTTTAACCCTGCTGGGTGCGG	62
K101E, V106M, G190A	V106M forward	V106M	GACAGTATTAGATGTGGGGGATGCA	64.4
	K101E, V106M reverse		ATTGATTTTTTCTTCTCTAACCCTGC	
K103S, V106M	K103S, V106M reverse		ATTGATTTGCTCTTTTTTAACCCTGC	58.3
K103N, V108I	K103N, V108I forward	V108I	ATTAGATGTGGGGGATGCATATTTTTC	63.2
K103N, V108I, P225H	K103N, V108I reverse		ATTGTTACTGATTTATTCTTTTTTAACCCTG	63.2
K103N, E138A	E138A forward	E138A	AATAGTCATCTATCAATATATGGATGATTTGTATG	59.6
	E138A reverse		GCTGGATTTTGTGTCCTAAAGGGCTC	
K103N, Y188L	Y188L forward	Y188L	AGTAGGATCTGATTTAGAAATAGGGC	54
V106M, Y188L	Y188L reverse		AACAAATCATCCATATATTGATAGATGACTATTTC	55
V179D, Y188L	V179D, Y188L reverse		AACAAATCATCCATATATTGATAGATGTCTATTTC	57
V106M, G190A	G190A forward	G190A	ATCTGATTTAGAAATAGGGCAACATAG	54
V106M, G190A, F227L	G190A reverse		GCTACATACAAATCATCCATATATTGATAGATG	54
V106M, V179D, F227L	F227L forward	F227L	ACTTTGGATGGGGTATGAACTCCATC	62.7
	F227L reverse		AATGGGGGTTCTTTCTGATGTTTCTTGTC	

* Some sets of primers were occasionally used to introduce more than one nucleotide change
** The nucleic bases that were changed to introduce mutations (■)
DRM – drug resistance mutation, T_m – melting temperature

Table S3: SDM primers used to generate single mutants in laboratory-adapted strains.

Mutation	Primer direction	Sequence (5' → 3')***	optimised T_m (°C)
V106M*	forward	CCACATCCAGTACTGTCATTGATTTTTTCTTTTTTAA	58.2
	reverse	TTAAAAAAGAAAAAATCAATGACAGTACTGGATGTGG	
V106M** in NSX	forward	GACAGTACTGGATGTGGGCG	59
	reverse	ATTGATTTTTTCTGTTTTAACCCTGCAGG	
F227L**	forward	ACTTTGGATGGGTTATGAACTCCATC	58
	reverse	AATGGAGGTTCTTTCTGATGTTTTTTGTC	

*SDM with overlapping primers was performed
**SDM with non-overlapping primers was performed
***The nucleic bases that were changed to introduce mutations are highlighted (■)
T_m – melting temperature

Table S4: IC_{50} values and FC for the single NNRTI mutations.

Mutation	Average IC_{50} (standard deviation) (µM)		Average Fold Change (standard deviation)		p-value**
**A98G**	0.017	(0.000)	2.99	(0.02)	**<0.0001**
**L100I**	0.008	(0.003)	1.36	(0.50)	0.188
**K101E**	0.010	(0.003)	1.79	(0.51)	0.114
**K101P**	0.005	(0.001)	0.85	(0.20)	0.465
**K103N**	0.005	(0.000)	0.96	(0.02)	0.274
**K103S**	0.007	(0.002)	1.23	(0.29)	0.300
**V106M**	0.097	(0.030)	17.33	(5.31)	**0.001**
**V108I**	0.016	(0.001)	2.84	(0.21)	**0.046**
**E138A**	0.009	(0.001)	1.56	(0.16)	0.119
**V179D**	0.004	(0.000)	0.64	(0.03)	**<0.0001**
**Y181C**	0.002	(0.000)	0.35	(0.07)	**0.014**
**Y188L**	0.500*	(0.000)	89.16	(0.00)	**<0.0001**
**G190A**	0.014	(0.003)	2.52	(0.55)	**0.041**
**P225H**	0.022	(0.009)	3.86	(1.60)	0.090
**F227L**	0.008	(0.001)	1.34	(0.24)	**0.031**
**MJ4**	0.006	(0.002)	1.00	(0.33)	-

*IC_{50} values exceed the maximum concentration tested which was 0.5 µM
**an unpaired Student's t-test test compared the Fold Change (FC) of each mutant to wild-type subtype C (MJ4) and the significant *p*-values are in bold
*** each mutant was tested in at least two independent assays in duplicate
IC_{50} – 50% inhibitory concentration

Table S5: IC_{50} values and FC for V106M in subtypes B and C.

Mutation	Laboratory Adapted Strain	Average IC_{50} (standard deviation) (µM)		Average Fold Change (standard deviation)		p-value*
**V106M**	**DS9**	0.044	(0.005)	13.40	(1.66)	**0.006**
	**LTNP5**	0.101	(0.009)	30.69	(2.75)	**0.003**
	**SM1**	0.068	(0.014)	20.55	(4.31)	**0.016**
	**SM2**	0.089	(0.019)	26.93	(5.63)	**0.015**
	**NSX**	0.042	(0.006)	12.69	(1.86)	**0.008**
	**NSX WT**	0.003	(0.003)	1.00	(0.33)	-
**V106M**	**CM9**	0.132	(0.013)	23.59	(2.36)	**0.004**
	**DU151**	0.043	(0.004)	7.73	(0.64)	**0.003**
	**DU179**	0.211	(0.051)	37.67	(9.02)	**0.020**
	**DU422**	0.109	(0.023)	19.46	(4.03)	**0.016**
	**MJ4**	0.097	(0.035)	17.33	(5.31)	**0.0007**
	**MJ4 WT**	0.006	(0.002)	1.00	(0.33)	-

* an unpaired Student's t-test test compared the Fold Change (FC) of each laboratory-adapted strain to the subtype wild-type (WT) reference and the significant *p*-values are in bold
** each laboratory strain was tested in at least two independent assays in duplicate
IC_{50} – 50% inhibitory concentration

Table S6: IC_{50} values and FC for F227L in subtypes B and C.

Mutation	Laboratory Adapted Strain	Average IC_{50} (standard deviation) (µM)		Average Fold Change (standard deviation)		p-value*
**F227L**	**DS9**	0.004	(0.0003)	1.30	(0.11)	**0.013**
	**LTNP5**	0.012	(0.003)	3.75	(1.01)	**0.040**
	**SM1**	0.008	(0.001)	2.45	(0.17)	**<0.0001**
	**SM2**	0.013	(0.001)	3.90	(0.35)	**0.002**
	**NSX**	0.003	(0.001)	0.97	(0.17)	0.824
	**NSX WT**	0.003	(0.003)	1.00	(0.33)	-
**F227L**	**CM9**	0.008	(0.001)	1.35	(0.11)	**0.023**
	**DU151**	0.005	(0.001)	0.91	(0.11)	0.290
	**DU179**	0.001	(0.000)	0.26	(0.07)	**<0.0001**
	**DU422**	0.004	(0.001)	0.69	(0.14)	0.053
	**MJ4**	0.008	(0.001)	1.34	(0.24)	**0.0307**
	**MJ4 WT**	0.006	(0.002)	1.00	(0.33)	-

* an unpaired Student's t-test test compared the Fold Change (FC) of each laboratory-adapted strain to the subtype wild-type (WT) reference and the significant *p*-values are in bold
** each laboratory strain was tested in at least two independent assays in duplicate
IC_{50} – 50% inhibitory concentration

Table S7: IC_{50} values and FC for combination NNRTI mutations.

Mutation	Average IC_{50}* (standard deviation) (µM)		Average Fold Change (standard deviation)		p-value**
**A98G,K103N**	0.045	(0.013)	8.1	(2.3)	**0.032**
**A98G,K103N,P225H**	0.451	(0.083)	80.5	(14.8)	**0.011**
**L100I,K103N**	0.035	(0.008)	6.3	(1.5)	**0.026**
**L100I,K103N,P225H**	0.164	(0.057)	29.3	(10.2)	**0.012**
**K101P,K103N**	0.005*	(0.001)	0.8	(0.1)	**0.104**
**K103N,V106M**	0.459	(0.070)	81.9	(12.5)	**0.008**
**K103N,V106M,F227L**	0.500*	(0.000)	89.2	(0.0)	**<0.0001**
**K103N,V108I**	0.016	(0.002)	2.8	(0.3)	**0.067**
**K103N,V108I,P225H**	0.098	(0.001)	17.5	(0.3)	**0.006**
**K103N,E138A**	0.007	(0.001)	1.2	(0.2)	**0.334**
**K103N,Y188L**	0.500*	(0.000)	89.2	(0.0)	**<0.0001**
**K103N,P225H**	0.054	(0.012)	9.7	(2.2)	**0.020**
**K101E,V106M,G190A**	0.500*	(0.000)	89.2	(0.0)	**<0.0001**
**K103S,V106M**	0.500*	(0.000)	89.2	(0.0)	**<0.0001**
**V106M,V179D**	0.037	(0.002)	6.7	(0.3)	**0.025**
**V106M,V179D,F227L**	0.500*	(0.000)	89.2	(0.0)	**<0.0001**
**V106M,Y188L**	0.500*	(0.000)	89.2	(0.0)	**<0.0001**
**V106M,G190A**	0.478	(0.038)	85.3	(6.7)	**0.002**
**V106M,G190A,F227L**	0.500*	(0.000)	89.2	(0.0)	**<0.0001**
**V179D,Y188L**	0.500*	(0.000)	89.2	(0.0)	**<0.0001**
**A98G,K103N**	0.006	(0.002)	89.2	(0.0)	**0.032**
**MJ4**	0.045	(0.013)	1.00	(0.3)	**0.011**

* IC_{50} values exceed the maximum concentration tested which was 0.5 µM
** an unpaired Student's t-test test compared the Fold Change (FC) of each mutation profile to wild-type subtype C (MJ4) and the significant *p*-values are in bold
*** each combination mutation was tested in at least two independent assays in duplicate
IC_{50} – 50% inhibitory concentration
